# Phenotypic Plasticity and Population Differentiation in an Ongoing Species Invasion

**DOI:** 10.1371/journal.pone.0044955

**Published:** 2012-09-19

**Authors:** Silvia Matesanz, Tim Horgan-Kobelski, Sonia E. Sultan

**Affiliations:** 1 Departamento de Biología y Geología, Universidad Rey Juan Carlos, Madrid, Spain; 2 Biology Department, Wesleyan University, Middletown, Connecticut, United States of America; University of Konstanz, Germany

## Abstract

The ability to succeed in diverse conditions is a key factor allowing introduced species to successfully invade and spread across new areas. Two non-exclusive factors have been suggested to promote this ability: adaptive phenotypic plasticity of individuals, and the evolution of locally adapted populations in the new range. We investigated these individual and population-level factors in *Polygonum cespitosum*, an Asian annual that has recently become invasive in northeastern North America. We characterized individual fitness, life-history, and functional plasticity in response to two contrasting glasshouse habitat treatments (full sun/dry soil and understory shade/moist soil) in 165 genotypes sampled from nine geographically separate populations representing the range of light and soil moisture conditions the species inhabits in this region. *Polygonum cespitosum* genotypes from these introduced-range populations expressed broadly similar plasticity patterns. In response to full sun, dry conditions, genotypes from all populations increased photosynthetic rate, water use efficiency, and allocation to root tissues, dramatically increasing reproductive fitness compared to phenotypes expressed in simulated understory shade. Although there were subtle among-population differences in mean trait values as well as in the slope of plastic responses, these population differences did not reflect local adaptation to environmental conditions measured at the population sites of origin. Instead, certain populations expressed higher fitness in both glasshouse habitat treatments. We also compared the introduced-range populations to a single population from the native Asian range, and found that the native population had delayed phenology, limited functional plasticity, and lower fitness in both experimental environments compared with the introduced-range populations. Our results indicate that the future spread of *P. cespitosum* in its introduced range will likely be fueled by populations consisting of individuals able to express high fitness across diverse light and moisture conditions, rather than by the evolution of locally specialized populations.

## Introduction

A key characteristic of invasive species is their ability to colonize and persist in a broad range of environmental conditions, including favorable, resource-poor and heterogeneous sites [1]–[5]. This ecological breadth may stem from two non-exclusive mechanisms: adaptive plasticity of individuals, and selective evolutionary change that adapts populations to diverse local environments [2], [6], [7].

Plasticity can play an important role in biological invasions by allowing individuals to colonize and establish in environmentally diverse habitats [1], [6], [8]. Specifically, functionally adaptive plasticity in allocational, morphological and physiological traits involved in resource acquisition can allow individuals to maximize reproductive plant fitness in different environments [8], [9]. For example, to functionally accommodate low light conditions, plants may express developmental modifications that maximize light interception (e.g. by increasing specific leaf area to produce large, thin leaves and elongating seedling internodes and stems, [10]–[16]). Likewise, to produce phenotypes that are functionally appropriate in high-light, moisture-limited conditions, individuals may respond by increasing assimilation rate per unit leaf area, allocating more biomass to roots and raising water use efficiency [17]–[22]. If phenotypic plasticity leads to functionally adaptive phenotypes in several environments, the same genotypes may be successful across a range of diverse habitats. Species composed of such adaptively plastic, generalist individuals are predicted to undergo limited selective divergence at the population level [23], [24], as has been shown empirically in several invasive plant taxa (e.g. [2], [3], [25]–[27]).

An invasive species may also spread across diverse habitats by undergoing local adaptation, evolving ecotypes with distinctive traits and/or patterns of individual plasticity [2], [3], [7], [28]. Introduced taxa may evolve rapidly in a new range as a result of founder effects, drift and hybridization [29]–[31] combined with novel selection pressures [32]–[35]. If sufficient heritable variation is available and alternative genotypes are favored in different habitats, natural selection in an introduced range may create a mosaic of locally adapted populations [5], [36], [37]. Such local adaptation can lead to increased local abundance and propagule pressure, and therefore can be a key mechanism of invasion success [3], [38]. Studies of several invasive plant taxa have found evidence for local adaptation in a range of morphological, physiological and life-history traits and plasticity patterns [28], [39]–[42], suggesting that the ecological breadth of some invasive species results from such adaptive differentiation.

Local adaptation and individual plasticity in invasive species can be investigated in controlled "common garden" experiments that simulate the diverse conditions found in natural habitats [3], [36], [43]. These studies provide evidence for local adaptation when genotypes from certain populations have higher overall fitness in conditions similar to the populations’ sites of origin than genotypes in populations from contrasting conditions [27], [36], [42], [44]. For example, genotypes from locally adapted shade populations would have higher fitness when grown in low-light conditions than genotypes from open, high-light sites. Population-specific patterns of plasticity for underlying life-history and functional traits can be examined as possible sources of such population-level fitness-environment correlations [3], [22]. If replicated at the genotype level, such studies can also reveal the extent to which individuals can succeed in contrasting conditions and their repertoires of adaptive plasticity [8]–[10], [23], [45]. Accordingly, studies that compare fitness and functionally important traits expressed by genotypes from different populations can provide insights to population and individual sources of adaptive diversity.

In this study, we investigated the relative roles of plasticity and local adaptation in an ongoing species invasion by comparing functional, life-history, and fitness responses in genotypes from nine populations of a newly invasive plant to two glasshouse treatments simulating contrasting habitats in the introduced range. *Polygonum* (s.l.) *cespitosum* Blume ( = *Persicaria cespitosa*, [46]) is a highly selfing annual introduced to North America from eastern Asia in the early 20^th^ century [47]. In its native range, and initially in northeastern North America, *P. cespitosum* was mainly restricted to shaded, moist habitats such as forest understories [48], [49]. In just the last two decades, the species has expanded in this introduced range to open habitats characterized by high light availability and potential soil moisture deficits [50], and it has recently been classified as an invasive in the region [51]. Because of its rapid, ongoing spread, *P. cespitosum* provides a compelling model system to examine the evolutionary strategies that allow a species to spread across diverse habitats in a new range.

**Figure 1:**
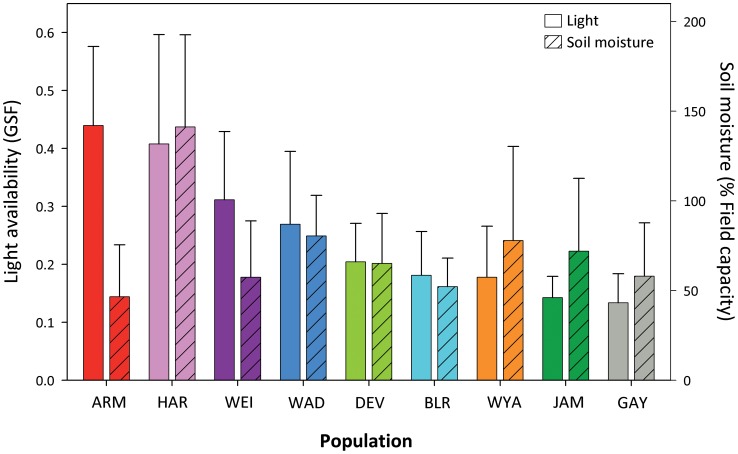
Light availability (global site factor) and soil moisture (% of field capacity) in the studied introduced range populations. Site means ± se are shown. Both light and water availability vary across as well as within populations sites (average coefficient of variation for each variable ranged from 0.36–0.58 across populations). Color coding of populations corresponds to that in figures 2–4. See text for measurement details.

We studied a set of nine populations that represent the current ecological distribution of *P. cespitosum* in northeastern North America. First, we determined environmental conditions in the populations’ source sites, which ranged from moderate and deep shade to full insolation, and flooded to dry soil moisture conditions (Fig. 1). We then grew highly inbred replicates of genotypes from all nine introduced-range populations in two glasshouse treatments designed to simulate the extremes of this ecological distribution: an understory, moist treatment and an open, dry treatment. We measured a suite of seedling, functional (morphological and physiological) and life-history and fitness traits in these plants to address the following specific questions: 1) Do introduced-range populations of the invasive *P. cespitosum* from diverse light and moisture environments show similar or different patterns of phenotypic plasticity in response to the contrasting glasshouse habitats? 2) If the populations differ, are these differences consistent with local adaptation, i.e., do populations perform best in conditions similar to their sites of origin? 3) We also compared the introduced-range populations to a single population sampled from the native range, to gain initial insight to possible differences in functional and fitness plasticity.

## Methods

### Study Populations

Field and herbarium records ([48]; The George Safford Torrey Herbarium, CONN, University of Connecticut) were used to identify *Polygonum cespitosum* populations in northeastern North America, where this species has been recently classified as invasive [51]. In October 2008, a set of 16 field populations that varied in light and soil moisture availability, disturbance level and habitat type was selected. From this initial set, a final group of 9 populations was chosen to represent the current ecological distribution of *P. cespitosum* in northeastern North America, based on the following criteria: i) the population was large, vigorous and well established, with >100 individuals covering at least 100 m^2^; ii) the site was not managed (e.g. mown or sprayed with herbicides); iii) the population did not adjoin highly disturbed areas (e.g. parking lots or buildings) and iv) the population was separated from other sample populations by at least 15 Km. (See Table S1 for population locations and habitat descriptions).

Light availability and soil moisture were characterized for all 9 populations at two timepoints during the *P. cespitosum* growth season (early July and September 2009). Light availability was quantified using hemispherical canopy photography, a widely used technique for exploring understory light conditions [52], [53]. A total of 15 hemispherical pictures were taken at each population on each date. Global site factor (GSF) was calculated for each population as the proportion of light reaching a site [54]. Soil moisture was calculated gravimetrically by extracting 10 soil cores (at two depths, 0–10 cm and 20–30 cm), from each of two transects covering the spatial extent of each population.

The 9 populations differ markedly in light availability and soil moisture (Fig. 1). This sample included (a) populations in forest understories where plants grew in the shade but received multiple sunflecks, i.e. moments of direct solar radiation [53], throughout the day (GAY, JAM and WYA); (b) temporally variable populations where plants received full sun during part of the day (ARM, DEV and WEI); and (c) spatially heterogeneous populations where shaded and full-sun microsites were present (BLR, HAR and WAD). Additionally, one population from a semi-shaded, moist site in the native range of the species (Japan) was included in the study (JPB; environmental data unavailable).

### Experimental Sample

We collected achenes from 35 field individuals in each population (25 in the native population) growing c. 1 m apart along linear transects, to insure a genotypic sample representing all microsites within each population. No specific permits were required for the described field studies in the introduced range, as the locations were not privately owned or protected in any way and there was no involvement of endangered or protected species. For the native population (JPB), we obtained all necessary permissions from the United States Department of Agriculture (permit number P37-08-01181) to import and grow small lots of seed. In March 2009, field-collected achenes were raised to maturity in uniform, favorable glasshouse conditions to produce inbred (selfed full-sib) genetic lines (hereafter genotypes) lacking any maternal-environment differences. Mature achenes were collected from each plant, air-dried and stored at 4°C.

Thirty-six achenes from each of 15–19 randomly selected genotypes per population (Table S1) were stratified in distilled water for 4 wk. at 4°C and sown into flats of moist vermiculite (8–10 June 2009). At the first true-leaf stage (5–7 July 2009), three replicate seedlings per genotype were randomly assigned to each of two glasshouse treatments (see below). Due to low germination in a few genotypes from the DEV and WYA populations and several missing replicates, the final sample was 840 plants [13–19 genotypes/population × 10 populations × 2 treatments × 3 replicates/treatment]. Seedlings from the native population (JPB) genotypes were assigned to the treatments 10 days later due to delayed germination; data collection and harvest for these plants were delayed accordingly.

### Experimental Habitat Treatments

Plants were grown for 10 wk. in two glasshouse treatments designed to mimic the extremes of the species’ current ecological distribution in its introduced North American range: Understory/Moist and Open/Dry (Figure 1). Seedlings were individually transplanted into 1 L clay pots filled with a 1∶1∶1 mixture of medium sand (Quickrete Co., Atlanta, GA USA), sterilized topsoil (Butler Construction, Portland, CT USA) and Turface MVP fritted clay (Profile, Buffalo Grove, IL USA), with 2.5 g per pot granular 15∶8∶12 NPK fertilizer (Agway, Middlefield, CT USA). All seedlings received 75% sun and ample moisture for 48 h, after which one replicate seedling per genotype was assigned to each treatment in each of three blocks (contiguous glasshouse compartments) in a complete randomized block design [55].

Plants in the Open/Dry treatment received full sun (mean midday PAR ∼1300 µmol m^−2^ s^−1^). Understory/Moist plants were grown under metal frames covered with neutral 80% shade cloth (PAK Unlimited Inc., GA USA) overlaid with green plastic filter strips (#138, Lee Filters, Burbank, CA USA) to simulate canopy shade [56]; mean midday PAR was c. 260 µmol m^−2^ s^−1^. To mimic forest understory conditions, we created sunflecks (transient spots of direct insolation that occur when sunlight passes through openings in the canopy, [57], [58]) by cutting equidistant 3.5 cm-diameter holes (one per pot) in the shade cloth. An extra row of holes was added along the frame edges to ensure that all pots received the same number of sunflecks. The metal frame was hung 35 cm above the bench and was situated so that the center of each pot received a ∼15 minute-sunfleck at noon. This sunfleck duration is typical of the shaded forest understories where *P. cespitosum* occurs (sunflecks lasting ≤15 minutes represent ∼90% of all sunflecks occurring in these sites; Horgan-Kobelski, Matesanz and Sultan, unpublished data).

Soil moisture was maintained by automatic systems that delivered reverse osmosis-filtered water to one watering tube per pot (Chapin Watermatics, Watertown, NY USA). Plants in the Open/Dry treatment received 10–15 ml 3–4 times a day for a mean soil moisture of 50% field capacity (9.23±0.44% moisture by mass, based on 3 soil samples from individual pots measured at four time points during the experiment, N = 12). Understory/Moist plants received 15–20 mL 3–4 times a day; soil moisture was 19.15±1.19%, (100% field capacity, N = 12).

### Data Collection

#### Seedling traits

On d15 in treatment, we measured seedling height (elongation above the cotyledons to the node of the most recent fully expanded leaf) and number of nodes in all seedlings; average internode length was calculated as seedling height/number of nodes.

#### Functional traits

Physiological performance: Physiological measurements were taken on all replicates for a subsample of 6–8 genotypes per population (N = 460). Data were collected between 9–14 h on 6 comparable sunny days (12–19 August); measurements were repeated (1 September) for 37 plants identified as outliers in a preliminary data analysis. *In situ* instantaneous photosynthetic rate and stomatal conductance were measured on 1 new, fully-expanded leaf of a primary branch per plant using a Li-Cor 6400 infrared gas analyser with red/blue LED light source and CO_2_ mixer (LI-COR, Lincoln, NE, USA). Instantaneous water use efficiency (iWUE) was calculated as the ratio of photosynthetic rate to stomatal conductance. Measurements were taken using a reference [CO_2_] of 400 µmol CO_2_ mol^−1^, PPFD of 1300 µmol m^−2^ s^−1^ in the Open/Dry treatment and 300 µmol m^−2 ^s^−1^ in the Understory/Moist treatment, stomatal ratio of 0.7 (L. Nichols, unpublished data) and gas flow of 500 µmol s^−1^. All plants were watered 30 minutes before measuring. Relative humidity was kept constant and close to ambient conditions (humidity range: 45–65%); air temperature ranged from 30–38°C. Measurements were logged only when the stability criteria were met (Licor 6400 User’s Manual, Li-COR Inc.). When the leaf did not completely cover the chamber, leaf tracings were scanned on a LI-3100 area meter (LI-COR, Lincoln, NE, USA) to determine photosynthetic surface area for rate calculations.

#### Allocation and morphology

After 10 wk. in treatment (September 17–22), aboveground tissues of each plant were harvested and separated, oven-dried (at 100°C for 1 h and then 65°C for ≥48 h) and weighed (leaf and stem biomass). Three non-senescent leaves from 1 primary branch per plant were scanned on an LI-3100 leaf area meter (LI-COR, Lincoln, NE, USA), oven-dried, and weighed to determine specific leaf area (SLA, leaf area/leaf biomass). Root systems were stored at 4°C before being manually washed, oven-dried (at 65°C for 48 h) and weighed to determine root biomass. Plant biomass was calculated as the sum of leaf, stem and root biomass. Whole-plant root to leaf biomass ratio was calculated (root: leaf biomass ratio).

#### Life-history and fitness traits

Reproductive onset for each plant (date of first flowering; defined by visible sepals of at least 1 flower) was determined through a daily census. Mature achenes were collected weekly during wk. 5–10. At final harvest (September 17–22), all remaining mature and immature achenes, flowers and reproductive support tissue were harvested. Achenes were air-dried for ≥5 d and weighed. Total reproductive output was calculated as the sum of the early maturing achenes plus all reproductive material collected at harvest. We also examined individual achene mass and achene number as components of total reproductive output. Mean individual achene mass was determined based on a random sample of 20 mature achenes per experimental plant (only 5–16 achenes were available for 50 individuals from the Understory/Moist treatment, and 30 individuals did not have mature achenes at the time of harvest). Achene number was estimated as total reproductive output/mean individual achene mass, and reproductive allocation was calculated as (total reproductive output/root + stem + leaf + reproductive mass) × 100%.

### Data Analyses

To assess differences between the introduced-range and the native population, mixed model ANOVA was used to test for the fixed effects of Population, Treatment and Block, the random effect of Genotype (nested within Population) and the interaction of Population and Treatment on all traits. To test the significance of effects, we used the Satterthwaite method of denominator synthesis, which finds the linear combinations of sources of random variation that serve as appropriate error terms for testing the significance of the respective effect [59]. Genotype × Treatment interaction was not included in the model as there was no replication (and therefore no variance) for a small number of genotypes due to missing replicates. Separate analyses conducted after removing those genotypes showed nearly identical results for trait means and plasticity. When the Population and/or Population × Treatment terms were significant, a planned (*a priori*) comparison of least squared means (linear contrast; [59]) was performed to compare the introduced-range populations to the native population within each treatment. Because additional comparisons are not performed, planned comparisons reduce the risk of inflating the type I error [55].

To analyze plasticity patterns and population differences in the introduced range, the mixed ANOVAs were repeated after removing the native population from the dataset. When the Population or Population × Treatment terms were significant, individual analyses were performed within each treatment to test for the fixed effect of Population and the random effects of Genotype (nested in Population) and Block. A significant effect of population was followed by post-hoc Student-Newman-Keuls (SNK) tests, performed on genotypic means [55]. Variables were log-transformed (achene number, and photosynthetic rate) or squared-root transformed (stem height, plant biomass, SLA and root: leaf ratio) to meet the assumptions of ANOVA [55]. Phenotypic selection analysis was used to assess selection on functional traits within each treatment. Selection differentials (*S’*), accounting for both direct and indirect selection on a trait, were calculated as the slope of the regression of each genotype’s standardized trait value against its relative fitness, estimated as mean total reproductive output [60], [61]. Genotypic trait means were used to reduce potential biases caused by micro-environmental covariance between individual trait values and fitness [62], [63]. Multivariate selection gradients [61] were not estimated because of high multicollinearity of the traits [21], [64], [65].

To test for local adaptation in the introduced-range populations, total reproductive output (mean of the population’s genotypic means) and mean values of functional traits in each treatment were regressed against light and soil moisture availability at each population site. A sequential Bonferroni correction was used to account for multiple comparisons [59]. Pearson’s correlation coefficients were also calculated between total reproductive output of each population in both treatments.

All data were excluded from 17 plants due to treatment or measurement error (final N = 823). Analyses were performed using Statistica 6.0 (Statsoft, Tulsa, OK USA).

## Results

### Individual Plasticity in Introduced-range Populations

Phenotypic expression for seedling traits, functional traits and fitness components was significantly affected by environmental treatment (Table 1; all Treatment effects significant at *P*<0.001). Except for the 3 physiological traits, these patterns of plastic response varied significantly among populations (Table 1, Population × Treatment terms). Although populations also differed on average for most traits, effects of environmental treatment were far greater (compare mean squares of Treatment and Population terms, Table 1). Seedling, life-history, and some functional traits also varied among genotypes within populations (Table 1, Genotype terms).

**Table 1:** Effects of environmental treatment, population and genotype on a) seedling traits, b) functional traits and c) fitness and life-history traits.

		Treatment	Population	Population × Treatment	Genotype (Population)	Block	Error
**a) Seedling traits**							
Stem height	MS	48.51	28.34	4.26	1.66	0.66	0.405
(*R^2^* = 0.65)	*F*	119.70***	18.92***	10.50***	4.07***	1.64 ns	
Mean internode length	MS	7.49	1.47	0.24	0.04	0.01	0.018
(*R ^2^* = 0.68)	*F*	424.01***	36.53***	13.83***	2.46***	0.69 ns	
**b) Functional traits**							
Root: Leaf Biomass ratio	MS	1769.47	4.35	3.36	0.60	1.55	0.441
(*R* ^2^ = 0.94)	*F*	3982.75***	8.36***	7.56***	1.35**	3.48*	
SLA	MS	20122.10	8.10	6.60	2.20	12.90	1.90
(*R* ^2^ = 0.94)	*F*	10792.2***	3.7***	3.6***	1.20^+^	6.9***	
Photosynthetic rate	MS	80156.00	82.00	56.00	88.01	252.00	38.0
(*R* ^2^ = 0.86)	*F*	2095.13***	0.94ns	1.47ns	2.29***	6.58**	
Stomatal conductance	MS	0.38	0.01	0.01	0.01	0.18	0.008
(*R* ^2^ = 0.23)	*F*	46.33***	0.62 ns	0.62 ns	1.43*	21.95***	
iWUE	MS	55909.40	129.30	105.50	203.00	3320.10	155.4
(*R^2^* = 0.54)	*F*	359.72***	0.64 ns	0.68 ns	1.31†	21.36***	
**c) Life-history and fitness traits**							
Total reproductive output	MS	129.87	0.27	0.10	0.04	0.36	0.0184
(*R^2^* = 0.92)	*F*	7061.45***	6.60***	5.52***	2.42***	19.43***	
Plant biomass	MS	84.67	0.08	0.39	0.05	0.69	0.029
(*R^2^* = 0.82)	*F*	2873.43***	1.76†	13.11***	1.58***	23.57***	
Reproductive allocation	MS	78881.40	561.60	292.00	71.30	354.70	19.0
(*R^2^* = 0.88)	*F*	4146.72***	8.609***	15.35***	3.75***	18.64***	
Reproductive onset	MS	41380.30	1630.30	400.20	219.60	104.30	28.5
(*R^2^* = 0.81)	*F*	1453.45***	8.28***	14.05***	7.72***	3.66*	
Individual achene mass	MS	23.48	2.50	0.08	0.03	0.27	0.012
(*R^2^* = 0.85)	*F*	1888.71***	80.04***	6.73***	2.75***	22.29***	
Achene number	MS	135.21	0.30	0.15	0.06	0.33	25
(*R^2^* = 0.90)	*F*	5337.03***	5.75***	5.92***	2.23***	12.87***	
	df	8	1	8	141(59)	2	

The model includes only introduced-range populations (JPB excluded). Adjusted model *R^2^*, Mean Square (MS), F-ratio (*F*), degrees of freedom (df) and significance levels are shown. Degrees of freedom for the model on the physiology traits are shown in parenthesis. ns, not significant;

† *P*<0.10;

* *P*<0.05,

** *P*<0.01,

*** *P*<0.001.

#### Seedling traits

**Figure 2:**
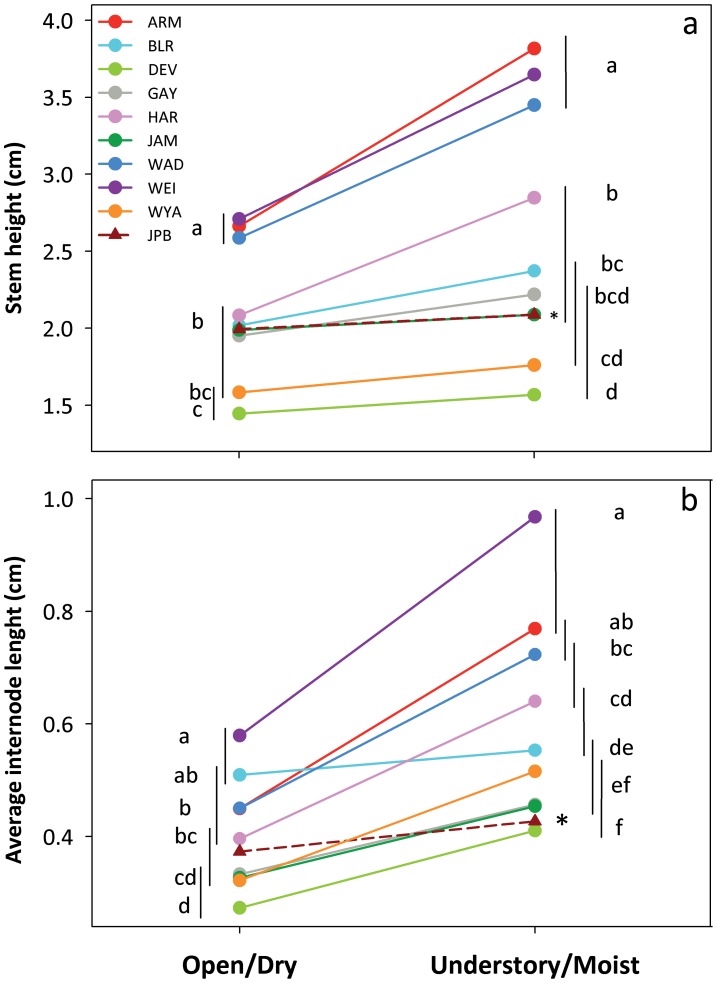
Population differences in plastic responses of seedling traits to contrasting habitat treatments. a) Stem height and b) Average internode length. Population means ± se are shown for 13–19 genotypes per population. Different letters and non-overlapping vertical lines show significant differences among populations in each habitat at the 0.05 level (SNK post-hoc test). An asterisk indicates a significant difference between the native population (JPB) and the introduced-range populations (linear contrast). Axes are scaled to 5–95% of data range.

Genotypes from all populations significantly increased stem height and average internode length in the Understory/Moist compared to the Open/Dry treatment (25% and 50% mean increase, respectively; Fig 2; Treatment effect, Table 1a). Populations varied on average and in the slope of these responses (Figure 2; Table 1a).

#### Functional traits

**Figure 3:**
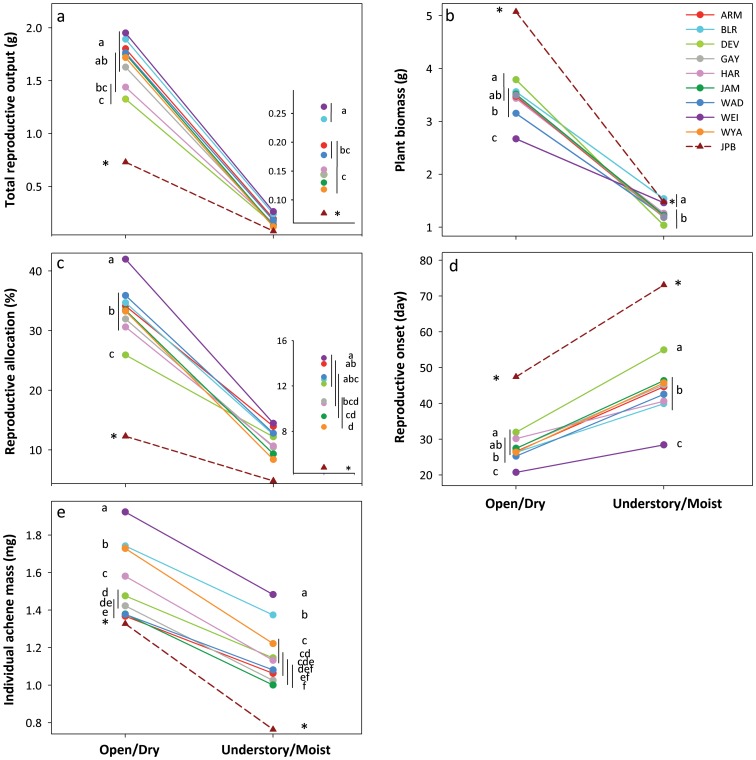
Population differences in plastic responses of morphological, allocational and physiological traits to contrasting habitat treatments. a) Specific leaf area, b) Root: Leaf Biomass ratio, c) Photosynthetic rate, d) Stomatal conductance and e) Water use efficiency. Population means ± se are shown for 13–19 genotypes per population. Inset in a), shows details of results in the Open/Dry treatment. Different letters and non-overlapping vertical lines show significant differences among populations in each habitat at the 0.05 level (SNK post-hoc test). An asterisk indicates a significant difference between the native population (JPB) and the introduced-range populations (linear contrast). NS indicates no significant population differences. Axes are scaled to 5–95% of data range.

Genotypes from all populations decreased specific leaf area (SLA) and increased root: leaf biomass ratio by c. 3-fold in the Open/Dry treatment compared with the Understory/Moist treatment (Fig. 3a–b). Although populations differed significantly in these traits within the Open/Dry treatment, they expressed similar phenotypes in the Understory/Moist treatment (Fig. 3a–b). Genotypes from all populations increased instantaneous photosynthetic rate (by 80%), stomatal conductance (27%) and water use efficiency (38%) in the Open/Dry treatment compared to the Understory/Moist treatment (Fig. 3c–e). These plastic responses were similar across all populations (NS effects of Population and Population × Treatment, Table 1b).

#### Life-history and fitness traits

**Figure 4:**
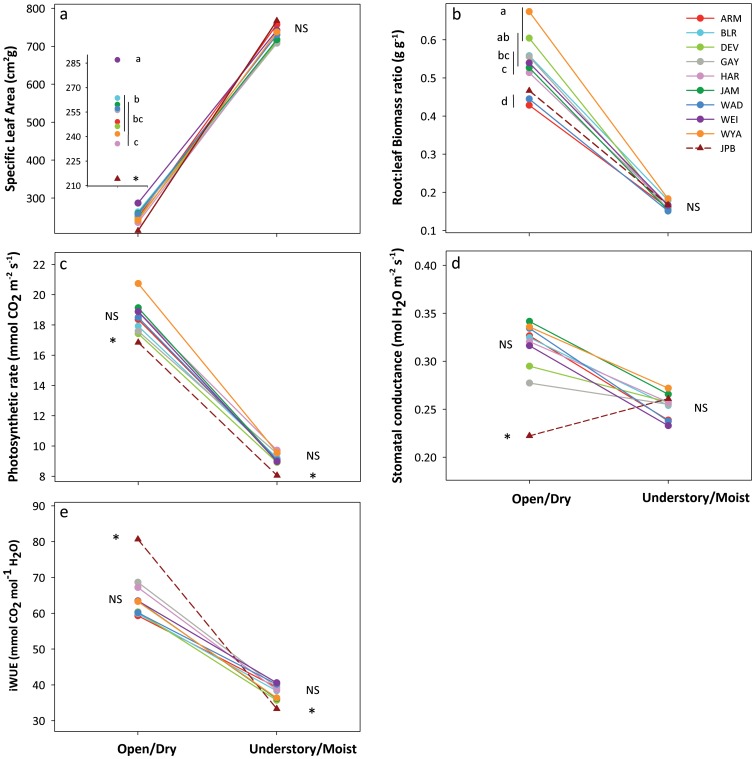
Population differences in plastic responses of life-history traits to contrasting habitat treatments. a) Total reproductive output, b) Plant biomass, c) Reproductive allocation, d) Reproductive onset and e) Individual achene mass. Population means ± se are shown for 13–19 genotypes per population. Insets in a), c) and f) show details of results in the Understory/Moist habitat. Different letters and non-overlapping vertical lines show significant differences among populations in each treatment at the 0.05 level (SNK post-hoc test). An asterisk indicates a significant difference between the native population (JPB) and the introduced-range populations (linear contrast). Axes are scaled to 5–95% of data range.

Genotypes from all populations significantly increased total reproductive output, reproductive allocation and plant biomass in the Open/Dry treatment compared to the Understory/Moist treatment (Fig. 4a–f). Increased reproductive output reflected increases in both achene number and individual achene mass. As above, in all cases the slope of these responses varied among populations, and average differences among introduced-range populations were small compared to the within-population effects of environment (Table 1c). On average, reproductive output was c. 10 times greater in the Open/Dry compared to the Understory/Moist treatment, and there was a 2–3-fold increase in reproductive allocation and plant biomass. Plants from all populations also showed faster reproductive onset in the Open/Dry treatment by an average of 16 days compared to inbred replicate plants in Understory/Moist conditions (Fig 4d).

### Tests of Local Adaptation in Introduced-range Populations

**Figure 5:**
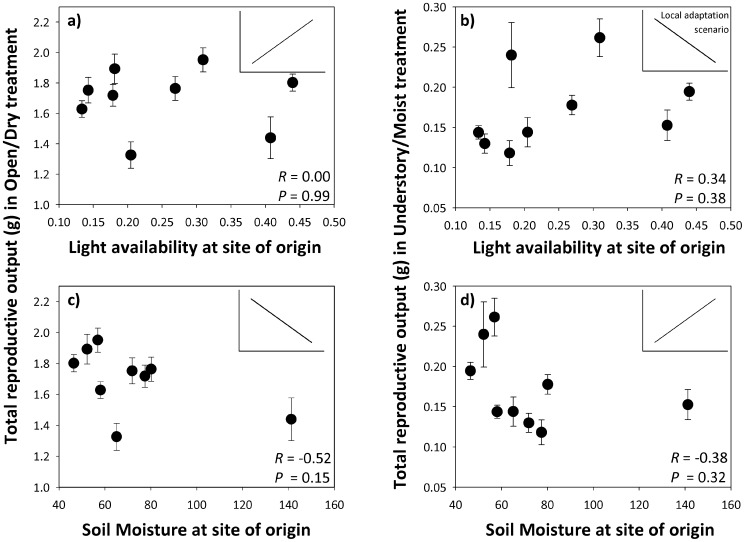
Lack of local adaptation in nine populations in the introduced range of *P. cespitosum*. Total reproductive output of each population (mean ± se) in each habitat treatment is plotted against light availability (measured as global site factor, top panels) and soil moisture (% of field capacity, bottom panels) at each population’s site of origin. Inset in each panel shows predicted pattern of local adaptation, i.e. higher reproductive output in the Open/Dry habitat treatment (left panels) in populations with (a) higher light availability and (b) lower soil moisture; higher reproductive output in the Understory/Moist habitat treatment (right panels) in populations with (c) lower light availability and (d) higher soil moisture. Tests of the reproductive output-environment correlations are shown (*R* and *P*-value); all are non-significant (0.15<p<0.99).

Correlations between reproductive output in experimental habitats and environmental conditions at the population sites of origin were non-significant; i.e. populations from sites with higher light availability and lower soil moisture did not have higher reproductive output in the Open/Dry treatment (Fig. 5 left), and populations from sites with lower light availability and higher soil moisture had no higher reproductive output in the Understory/Moist treatment (Fig. 5 right). Correlations between light and soil moisture availability and all other traits were also non-significant, after correcting for multiple comparisons (lowest *P = *0.018 for 13 correlations).

**Figure 6:**
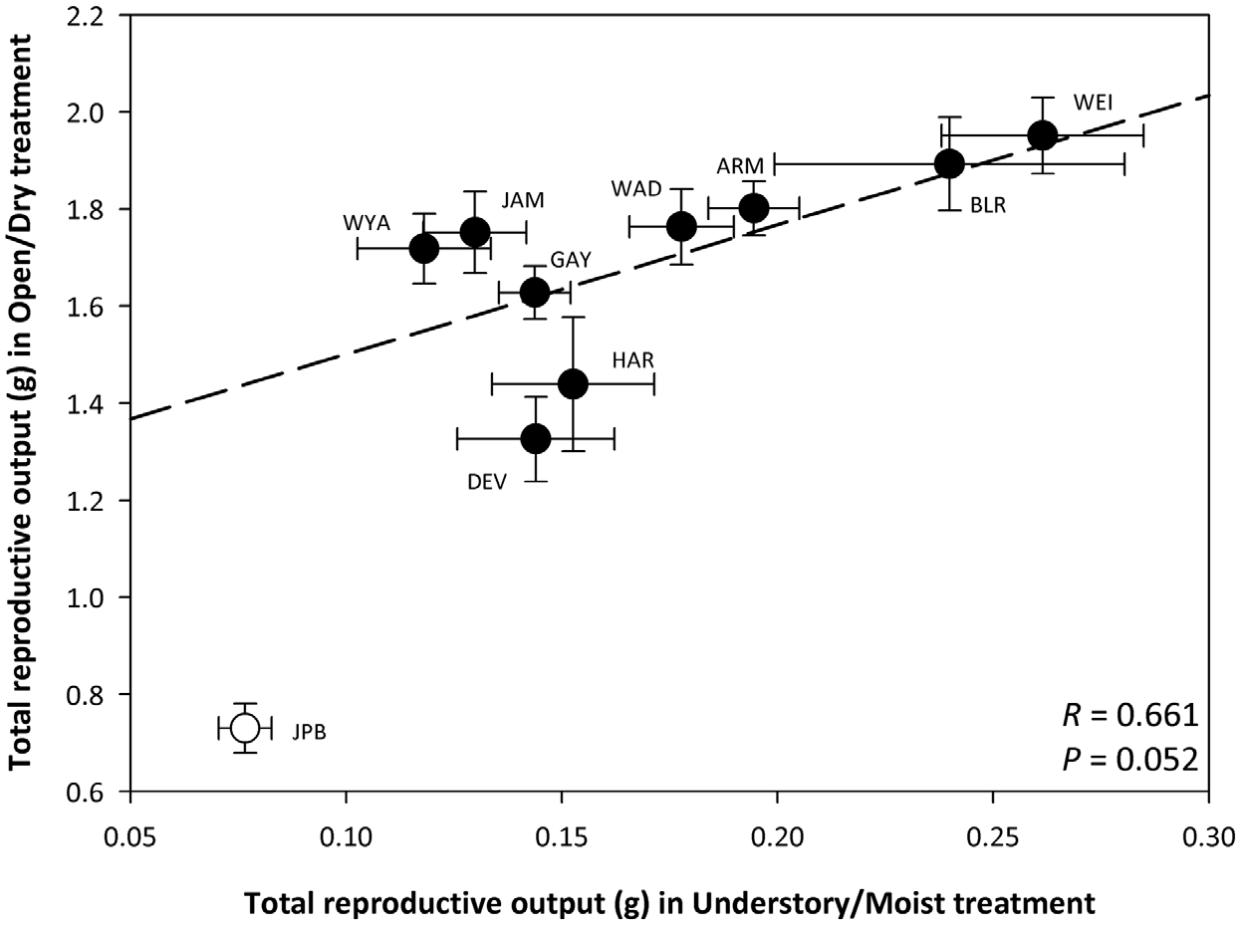
Correlation between total reproductive output in Open/Dry treatment and Understory/Moist treatment. Means (± se) are shown for each population. Regression line, correlation coefficient (*R*) and *P*-value are shown. The native population (JPB, open circle) was excluded from the analysis. Five out of the 9 populations (WEI, BLR, ARM, WAD and GAY) ranked in the same position in both treatments.

No fitness trade-off (negative correlation) was found for reproductive output in the two habitat treatments (Fig. 6). Instead, populations with relatively high reproductive output in the Open/Dry treatment also had high reproductive output in the Understory/Moist treatment, and *vice versa* (marginally significant positive correlation; Fig. 6). The same pattern was found when populations were pooled and genotypic means were used (Pearson’s *R* = 0.30, *P*<0.0001).

### Phenotypic Selection Analyses

Greater stem height, internode length, reproductive allocation, and early reproductive onset were associated with fitness in both treatments (Table 2). Fitness associations differed for SLA (positively associated with fitness only in Open/Dry conditions), root: leaf ratio (positively associated with fitness in Understory/Moist conditions) and biomass (positively associated with fitness only in Understory/Moist conditions Table 2). No significant association between fitness and physiological traits was found in either treatment.

**Table 2:** Results of the Phenotypic Selection Analyses.

	Open/Dry	Understory/Moist
	*S′*	*S′*
**a) Seedling traits**		
Stem height	0.0838***	0.2227***
Avg. Internode length	0.0868***	0.2097***
**c) Functional traits**		
SLA	0.0765***	−0.0529^ns^
Root to leaf ratio	−0.0334†	0.1305**
**b) Life-history and fitness traits**		
Reproductive allocation	0.1720***	0.4401***
Reproductive onset	−0.1244***	−0.2742***
Plant biomass	−0.0050^ns^	0.3065***

Standardized linear selection differentials (S’) are shown. See text for details on the analyses. ns, not significant;

† P<0.10;

* P<0.05,

** P<0.01,

*** P<0.001.

### Comparisons of Introduced-range Populations to the Native Population

Linear contrasts revealed significant differences between the native population and the introduced range populations for most fitness and functional traits (asterisks, Figs. 2–4). In both treatments, reproductive allocation and output were 50–60% lower in genotypes from the JPB population than in those from introduced-range populations (Table S2; Fig. 4). On average, plants from the JPB population produced 50% fewer achenes, of 15% lower mass, in the Open/Dry treatment, and a similar number of 38% lower-mass achenes in the Understory/Moist treatment; they delayed reproductive onset by 20 d and 30 d, respectively, in the two treatments (Table S2; Fig. 4). Conversely, biomass of JPB plants averaged 50% and 20% higher than that of introduced-range plants in the Open/Dry and Understory/Moist treatment, respectively. Although JPB plants had lower SLA in the Open/Dry treatment, SLA in Understory/Moist conditions and root: leaf ratio in both treatments were similar to introduced-range populations (NS contrasts; Table S2; Fig. 4). Genotypes from the JPB population had ∼10% lower photosynthetic rate in both treatments. Unlike genotypes from all of the introduced-range populations, JPB genotypes decreased conductance in the Open/Dry treatment, resulting in 22% higher iWUE than introduced-range populations (Table S2; Fig. 3).

## Discussion

### Phenotypic Plasticity in Introduced-range Populations

Despite pronounced site-of-origin differences in light and soil moisture availability, *Polygonum cespitosum* genotypes from introduced-range populations expressed broadly similar patterns of fitness and functional plasticity in two contrasting habitat treatments. Plants from all nine populations were able to exploit the high resource levels of the high-light, dry treatment by sharply increasing physiological performance and allocation to root tissues and reducing specific leaf area, resulting in a dramatic increase in reproductive fitness compared to plants in simulated understory shade. Conversely, these genotypes responded to low-light, moist conditions by increasing seedling elongation and specific leaf area and reducing root allocation relative to leaf tissue. These developmental and physiological adjustments are well-understood functionally adaptive responses to moisture and light limited conditions, respectively (see e.g. [10]–[12], [14]–[19], [21], [22], [66]). Furthermore, all genotypes from these populations were able to survive and produce viable offspring in both of these dramatically different, stressful environments. Such high plasticity and associated environmental tolerance are characteristic of widespread colonizing annuals and can contribute to invasion success by allowing species to successfully establish in diverse habitats [1], [2], [6], [9], [45], [67].

Although phenotypic responses of plants to contrasting light and moisture conditions were consistent with ecophysiological expectations, the phenotypic selection analyses did not provide statistical support for their adaptive value in all instances. For example, total reproductive output was not associated with either greater root allocation in open, dry conditions or increased specific leaf area in shaded, moist conditions. Contrary to expectations, high specific leaf area was favored in open, dry conditions and high root: leaf ratio was favored in the understory, moist treatment. These unexpected results for traits of clear functional importance suggest that relatively subtle differences among genotypes in these traits may be offset by differences in other related, unmeasured traits [68]. For example, the association of high specific leaf area with reproductive fitness in the high-light, dry treatment could reflect the ability of these plants to maintain a large transpiring surface area by modifying functionally related aspects of phenotypic expression such as root morphology or uptake physiology [69]. Similar, counter-intuitive results were reported by Gianoli and González-Teuber [70], who found that high leaf area was positively associated with fitness in dry conditions in a perennial herb (see e.g. [68], [71] for other studies where phenotypic selection analyses do not match ecophysiological predictions). Furthermore, because selection differentials reflect both direct and indirect selection on a trait, positive associations of these traits with fitness may be due to genetic correlations among traits rather than to direct selection on the specific trait [22], [72]. These results highlight both the complex contributions of correlated functional traits to fitness, and the interpretive limitations of statistical approaches to testing adaptation [22], [61], [63], [73], [74].

### Lack of Local Adaptation to Introduced-range Habitats

Though patterns of plastic response were generally similar across populations, there were (comparatively subtle) differences between populations in both mean trait values and response slopes for most reproductive and functional traits. However, these population differences evidently did not reflect adaptation to local light and soil moisture conditions. Lack of local adaptation can be due to low genetic variation constraining evolutionary change, as has been found in other invasive species (e.g. [3], [27]). However, the lack of local adaptation in this system is likely not due to genetic constraints, given the ample quantitative genetic variation here documented for most traits, and the evidence that other introduced-range populations of *P. cespitosum* have shown rapid evolutionary change [50]. Our results concur with studies of several invasive plants that show both high plasticity and lack of local adaptation in introduced-range populations (e.g. [2], [3], [25]–[27]). It is also possible that these populations are locally adapted to a less obvious environmental factor that was not measured.

Contrary to the fitness trade-off expected under a pattern of local adaptation, the mean reproductive output of *Polygonum* populations was positively correlated between environments, i.e. populations with relatively high fitness in open, dry conditions also had high fitness in understory, moist conditions. The relatively small yet consistent fitness differences among populations across treatments suggest that certain high-fitness populations may contribute disproportionately to the spread and further evolution of *P. cespitosum* in the introduced range. These results provide empirical support to the concept that invasion dynamics may be strongly influenced by population-level differences ([75], Matesanz and Sultan, submitted ms.).

### Generalist Adaptation

These patterns of individual plasticity and population differentiation indicate that instead of local specialists, this newly invasive species consists of highly plastic, generalist populations that can successfully establish in diverse sites [76], [77]. Depending on the scale of environmental heterogeneity, adaptive evolution in an introduced range can lead to generalist genotypes rather than local ecotypes (e.g. [78]–[82]). Alternatively, high performance across contrasting conditions in an introduced range can result from the introduction of preadapted genotypes that evolved generalist adaptations in the native range [32], [83]. Although the single native-range population in our sample is insufficient to provide a robust test (and indeed, even the largest sample cannot falsify the hypothesis that preadapted genotypes may have evolved in a native-range population), the striking differences between the introduced-range and native populations of *P. cespitosum* suggest that generalist genotypes may have evolved subsequent to the species’ introduction to North America. This possibility is supported by the results of a resurrection experiment documenting the recent evolution in introduced-range populations of this species of increased photosynthetic rates and reproductive output in open conditions [50].

The native population we studied produced higher biomass but lower reproductive allocation (and both fewer and smaller offspring) in both glasshouse habitat treatments. This population also showed a distinct physiological behavior characterized by low photosynthetic rates and reduced conductance in response to high light. This response pattern is consistent with the distribution of *P. cespitosum* in its native range, where the species does not occur in full-sun, dry sites ([49], Z. Kikvidze, personal communication). However, we emphasize that a single population cannot be assumed to represent the entire species in its native range. Further, studies that include a broader sample of native-range populations will help to determine whether the generalist nature of plants in the introduced range is due to *in situ* adaptive evolution.

Whether these broadly plastic *P. cespitosum* genotypes evolved prior to or after the species’ introduction to North America, certain seedling and life-history traits are associated with enhanced fitness in both of the contrasting light and moisture environments we studied. As shown by the phenotypic selection analyses, genotypes from populations with greater average fitness showed faster seedling growth, earlier reproductive onset and higher reproductive allocation in both open, dry and understory, moist glasshouse environments. These traits are likely to confer a competitive advantage under natural conditions [7], [84] and have been consistently reported in generalist invasive plants (e.g., [79], [85]). These results, along with patterns of individual plasticity that contribute to successful function in contrasting conditions, suggest that the spread of this newly invasive plant across diverse habitats in its introduced range reflects the adaptive scope of generalist genotypes rather than the evolution of locally adapted, functional specialists.

## Supporting Information

Table S1 Location, habitat description and number of genotypes of 9 populations of *Polygonum cespitosum* from the introduced range (Northeastern North America) and one population from the native Asian range.
(DOCX)

Table S2 Results of the linear contrasts (*F*-ratio and *P*-value) comparing a native population (JPB) to nine introduced-range populations. † P<0.10; * P<0.05, ** P<0.01, *** P<0.00. See text for details on the statistical analyses.
(DOC)
