# Supplementary material for: Phenotypic Plasticity and Population Differentiation in an Ongoing Species Invasion
Source: PLoS One. 2012 Sep 19;7(9):e44955. doi: 10.1371/journal.pone.0044955 (PMC3446995; doi:10.1371/journal.pone.0044955)
Supplement: Table S1 — Location, habitat description and number of genotypes of 9 populations of Polygonum cespitosum from the introduced range (Northeastern North America) and one population from the native Asian range. (DOCX) [file pone.0044955.s001.docx]

**Table S1**. Location, habitat description and number of genotypes of 9 populations of *Polygonum cespitosum* from the introduced range (Northeastern North America) and one population from the native Asian range.

|  | **Code** | **Population location** | **Geographical coordinates** | **Type of habitat** | **Number of genotypes** |
| --- | --- | --- | --- | --- | --- |
| **Introduced range** |  |  |  |  |  |
|  | **ARM** | Arch Road, Leeds, MA | 42º21’13’’N, 72º41’39’’W | Roadside | 17 |
|  | **BLR** | Black Rock State Park, Thomaston, CT | 41º39’24’’N, 73º06’18’’W | Trailhead and forest edge | 15 |
|  | **DEV** | Devils Hopyard State Park, East Haddam, CT | 41º28’42’’N, 72º20’30’’W | Roadside | 13 |
|  | **GAY** | Gay City State Park, Hebron, CT | 41º43’47’’N, 72º26’20’’W | Forest trail | 19 |
|  | **HAR** | Harvard Arnold Arboretum, Jamaica Plain, MA | 42º18’08’’N, 71º07’27’’W | Lowland clearing | 17 |
|  | **JAM** | James Goodwin State Forest, Hampton, CT | 41º46’40’’N, 72º05’12’’W | Forest horse trail | 19 |
|  | **WAD** | Wadsworth Estate, Middletown, CT | 41º32’07’’N, 72º40’33’’W | Forest horse trail and clearing | 19 |
|  | **WEI** | Weir Farm, Wilton, CT | 41º15’23’’N, 73º27’22’’W | Roadside | 18 |
|  | **WYA** | Wyantenock State Forest, Kent, CT | 41º45’47’’N, 73º23’52’’W | Forest trail | 13 |
|  |  |  |  |  |  |
| **Native range** | **JPB** | Inage, Chiba Prefecture, Japan | 35º38’08’’N, 140º05’02’’E | Forest edge and adjacent meadow | 16 |
