# Supplementary material for: Phenotypic Plasticity and Population Differentiation in an Ongoing Species Invasion
Source: PLoS One. 2012 Sep 19;7(9):e44955. doi: 10.1371/journal.pone.0044955 (PMC3446995; doi:10.1371/journal.pone.0044955)
Supplement: Table S2 — Results of the linear contrasts (F-ratio and P-value) comparing a native population (JPB) to nine introduced-range populations. † P<0.10; * P<0.05, ** P<0.01, *** P<0.00. See text for details on the statistical analyses. (DOC) [file pone.0044955.s002.doc]

**Table S2**. Results of the linear contrasts (*F*-ratio and *P*-value) comparing a native population (JPB) to nine introduced-range populations. † P < 0.10; * P < 0.05, ** P < 0.01, *** P < 0.00. See text for details on the statistical analyses.

|  | **Open/Dry** | **Understory/Moist** |
| --- | --- | --- |
| **a) Seedling traits** |  |  |
| Stem height | 0.20ns | 18.3*** |
| . |  |  |
| Mean internode length | 0.77ns | 45.48*** |
| **b) Functional traits** |  |  |
| Root: Leaf Biomass ratio | 1.98ns | 1.24ns |
|  |  |  |
| SLA | 37.4*** | 1.96ns |
|  |  |  |
| Photosynthetic rate | 5.2* | 36.2*** |
|  |  |  |
| Stomatal conductance | 10.9** | 0.46ns |
|  |  |  |
| iWUE | 7.6** | 17.97*** |
| **c) Life-history and fitness traits** |  |  |
| Total reproductive output | 221.6*** | 57.9*** |
|  |  |  |
| Plant biomass | 157.4*** | 15.15*** |
|  |  |  |
| Reproductive allocation | 775.9*** | 124.9*** |
|  |  |  |
| Reproductive onset | 1457.4*** | 644.1*** |
|  |  |  |
| Individual achene mass | 175.1*** | 113.8*** |
|  |  |  |
| Achene number | 111.2*** | 0.2ns |
